# Outcomes and toxicities in oligometastatic patients treated with stereotactic body radiotherapy for adrenal gland metastases: A multi-institutional retrospective study

**DOI:** 10.1016/j.ctro.2021.09.002

**Published:** 2021-10-26

**Authors:** A. Baydoun, H. Chen, I. Poon, S. Badellino, R. Dagan, D. Erler, M.C. Foote, A.V. Louie, K.J. Redmond, U. Ricardi, A. Sahgal, T. Biswas

**Affiliations:** a Department of Radiation Oncology, University Hospitals of Cleveland, Cleveland, OH 44106, USA; b Department of Radiation Oncology, Sunnybrook Health Sciences Centre, Toronto, Ontario M4N 3M5, Canada; c Radiation Oncology Unit, Department of Oncology, University of Turin and Città della Salute e della Scienza Hospital, Via Genova 3, Turin 10126, Italy; d Department of Radiation Oncology, University of Florida Health Proton Therapy Institute, Jacksonville, FL 32206, United States; e Department of Radiation Oncology, Princess Alexandra Hospital, Woolloongabba, QLD 4120, Australia; f Department of Radiation Oncology and Molecular Radiation Sciences, The Johns Hopkins University, Baltimore, MD 21218, United States

**Keywords:** Adrenal gland metastases, SBRT, Oligometastases

## Abstract

**Background:**

Studies reporting SBRT outcomes in oligometastatic patients with adrenal gland metastases (AGM) are limited. Herein, we present a multi-institutional analysis of oligometastatic patients treated with SBRT for AGM.

**Material/methods:**

The Consortium for Oligometastases Research (CORE) is among the largest retrospective series of patients with oligometastases. Among CORE patients, those treated with SBRT for AGM were included. Clinical and dosimetric data were collected. Adrenal metastatic burden (AMB) was defined as the sum of all adrenal GTV if more than one oligometastases is present.

Competing risk analysis was used to estimate actuarial cumulative local recurrence (LR) and widespread progression (WP). Kaplan-Meier method was used to report overall survival (OS), local recurrence-free survival (LRFS), and progression-free survival (PFS). Treatment related toxicities were also reported.

**Results:**

The analysis included 47 patients with 57 adrenal lesions. Median follow-up was 18.2 months. Median LRFS, PFS, and OS were 15.3, 5.3, and 19.1 months, respectively. A minimum PTV dose BED_10_ > 46 Gy was associated with an improved OS and LRFS. A prescribed BED_10_ > 70 Gy was an independent predictor of a lower LR probability. AMB>10 cc was an independent predictor of a lower risk for WP. Only one patient developed an acute Grade 3 toxicity consisting of abdominal pain.

**Conclusion:**

SBRT to AGM achieved a satisfactory local control and OS in oligometastatic patients. High minimum PTV dose and BED_10_ prescription doses were predictive of improved LR and OS, respectively. Prospective studies are needed to determine comprehensive criteria for patients SBRT eligibility and dosimetric planning.

## Highlights

• SBRT to adrenal gland oligometastases achieves a satisfactory local control and OS.
• A minimum PTV dose BED_10_ > 46 Gy was associated with an improved OS and LRFS.
• A prescribed BED_10_ > 70 Gy was correlated with improved local control.
• High adrenal metastases volume should not preclude the delivery of SBRT.

## 1 Introduction

Since its first use in the treatment of extracranial tumors in the early 1990s, stereotactic body radiotherapy (SBRT) has been explored and validated in a myriad of disease sites and settings [1]. Particularly in the context of oligometastic disease defined as a state of limited systemic metastatic foci [2], SBRT provides the dosimetric and practical advantages of delivering high doses with ablative potential, in five or fewer fractions [3]. As such, SBRT was incorporated in the treatment scheme of patients with oligometastases (OM) as a non-invasive and well-tolerated alternative to surgical resection [3]. For example, multiple phase I and II studies reported improved local control rates with the use of SBRT to treat metastatic lesions in the lung [4], [5], liver [4], [6], spine [7], and multiple other sites [8], [9], [10], including the adrenal gland [8].

In fact, adrenal gland metastases (AGM) are the most common malignant lesions involving the adrenal glands [11]. While historically reported on post mortem autopsies [11] with a prevalence rate of 3.1% [12], the wide access to three dimensional anatomical imaging via computed tomography (CT), positron emission tomography, and magnetic resonance (MR) imaging resulted in an increased rate of AGM diagnosis [13]. Particularly in patients with oligometastatic primary malignancy, the incidence of AGM is between 1.5 and 3.5% [14], with non-small-cell lung cancer (NSCLC), renal cell carcinoma (RCC), and melanoma being the most common primaries [15]. From the other hand, many of the oligometastatic patients with AGM are not candidate for adrenalectomy [16] due to limited performance status [17], the presence of more than one metastatic site [16], and to the associated sub-optimal prognosis [17]. Therefore, SBRT has emerged as an attractive substitute for adrenalectomy with promising outcomes. Nevertheless, no specific guidelines are yet established to guide patients’ inclusion and dosimetric planning.

To date, most of the available literature on SBRT outcomes for AGM comes from either from single institutional experience [18], or from few published multi-institutional studies where a significant percentage of patients was treated with rather a palliative approach [19], or had a non-oligometastatic disease status [20]. Given the paucity of the existing data, we present in this manuscript the results of a multi-institutional analysis for patients with oligometastatic cancer treated with SBRT for their AGM. In addition to reporting AGM SBRT outcomes, the purpose of this study was to investigate possible association between outcomes and patients' dosimetric and clinical attributes.

## 2 Materials and methods

### 2.1 Patients selection

The Consortium for Oligometastases Research (CORE) is one of the largest retrospective series of patients with OM with>1000 patients included. The consortium’s establishment was detailed in a previous publication [21]. Briefly, it includes 1033 adult patients who underwent 1416 SBRT courses at six high-volume academic radiation oncology centers [21]. OM state was defined as the development of five or fewer extracranial metastases, synchronously (within six months of diagnosis) or metachronously (more than six months after diagnosis). Patients with oligoprogressive disease from previously widespread metastases prior to enrollment were excluded. However, once a patient was enrolled, subsequent oligorecurrent lesions treated with SBRT were captured in our database. Patients were also classified using the European Society for Radiotherapy and Oncology (ESTRO) and European Organisation for Research and Treatment of Cancer (EORTC) consensus classification [22]. SBRT was defined as the radiation course administered to the OM in 15 fractions or less, for definitive treatment intent. Patients with OM who were treated for palliative intent with fractionation schemes were excluded [21].

Among CORE patients, those treated with SBRT for AGM were identified and included in this study.

### 2.2 Treatment and Follow-up

Institutional protocols for simulation, immobilization, treatment planning, and image guidance were performed based on an international SBRT for OM consensus reported in 2017 [23]. After completion of SBRT, patients were followed-up longitudinally every 2–4 months in the first year, every 3–6 months in the second year, every 4–6 months in the third and fourth year, and every 6–12 months thereafter [21].

### 2.3 Statistical analysis

For all patients, baseline demographic and clinical characteristics were collected. These included age, gender, primary site, synchronous versus metachrnous state, total number of OM, status of the primary tumor, and pre- and post-SBRT systemic therapy. Dosimetric data including treatment volumes, prescribed dose, and number of fractions were also gathered. All doses were transformed to BED_10_, with an alpha/beta = 10. A student’s *t*-test was performed to compare the prescription BED_10_ dose for those treated for a single versus multiple AGMs. Competing risk analysis [24] was used to estimate the actuarial cumulative local recurrence (LR) over time and widespread progression (WP) over time, using death from any cause as a competing risk factor. Kaplan-Meier method [25] was used to report the overall survival (OS), local recurrence-free survival (LRFS), and progression-free survival (PFS). Univariable competing risks regression analysis with Fine and Gray method [26] was used for LR and WP, whereas Cox regression analysis [27] was used for LRFS, PFS, and OS. Variables that passed the univariable screen (p-value less than 0.15) were entered into multivariable models and backward selection was used to generate parsimonious models using a p-value threshold of 0.05. Cases with missing covariates were excluded from regression analyses. To account for the missing gross tumor volume (GTV) of some AGM, we defined the GTV/Internal target volume (ITV) variable as being the GTV if GTV is available, and the ITV if not. We also defined the adrenal metastatic burden (AMB) as being the sum of the patient’s all adrenal GTV/ITV. The start date for time-to-event analysis was defined as the end date for the first course of SBRT treatment. Statistical analyses were performed using R, version 4.0.2 [28].

## 3 Results

A detailed report of the clinical and dosimetric variables and statistical analysis is attached as an appendix to this article.

### 3.1 Patients and lesions characteristics

A total of 47 patients with 57 adrenal lesions were included in this study. The follow-up duration ranged between 2.0 and 67.0 months, with a median of 18.2 months and an interquartile range of 9.9–30.5 months. The distribution of the patients and lesions are summarized in Table 1.

**Table 1:** Patient and Lesions Characteristics.

**Variable**	Patients	AGM
Number	47	57
Primary Site (nb (%))		
● Colorectal	3 (6.4)	3 (5.3)
● Hepatocellular	1 (2.1)	1 (1.8)
● Kidney	4 (8.5)	5 (8.8)
● Melanome	1 (2.1)	1 (1.8)
● NSCLC	30 (63.8)	36 (63.2)
● Prostate	1 (2.1)	1 (1.8)
● Sarcoma	2 (4.3)	3 (5.3)
● SCLC	4 (8.5)	6(10.5)
● Stomach	1 (2.1)	1 (1.8)
Histology (nb (%))		
● Adenocarcinoma	26 (55.3)	30 (52.6)
● Clear cell	4 (8.5)	5 (8.8)
● Small Cell	4 (8.5)	6 (10.5)
● Squamous Cell Carcinoma	6 (12.8)	7(12.3)
● Other	7 (14.9)	9 (15.8)
Total Number of OM per patient (nb (%))		N/A
• 1	26 (55.3)	
• 2	14 (29.8)	
• 3	5 (10.6)	
• 4	0 (0)	
• 5	2 (4.2)	
Metastasis Timing		N/A
• Synchronous	21 (44.7)	
• Early metachronous (6–24 months)	5 (10.6)	
• Late metachronous (>24 months)	21 (44.7)	
Guckenberger et al. Classification		N/A
• Synchronous Oligometastatic	21 (44.7)	
• Metachronous	16 (34.0)	
• Oligorecurrence		
• Metachronous oligoprogression	8 (17.0)	
• Repeat Oligorecurrence	2 (4.3)	

The mean (standard deviation (SD)) age at diagnosis was 65.8 (10.8) years, with 25 (53.2%) male patients and 22 (46.8%) female patients. NSCLC was the most common primary malignancy, with 30 NSCLC patients presenting with a total of 36 OM. The most common primary histology was adenocarcinoma, and it accounted for 26 patients with 30 OM. The primary tumor exhibited local control for 41 (87.3%) patients, and local failure for six (12.8%) patients. Two of the patients included in this study had more than three OM in total. Nine (19.1%) patients received more than one course of AGM SBRT: seven were treated concurrently for two AGM only, one patient was treated for one AGM at the time of inclusion and later a second oligorecurrent AGM, and one patient was treated for two AGM initially then later for an oligorecurrent AGM. No statistically significant difference (p = 0.16) in prescription BED_10_ was found for those treated for a single AGM versus those treated with two or more AGMs. Among the 47 patients, 31 (66.0%) had received no systemic therapy prior to SBRT, whereas 12 (25.5%), 2 (4.3%), and 2 (4.3%) had received cytotoxic chemotherapy, immunotherapy, and targeted therapy prior to SBRT, respectively.

The dosimetric variables, including motion management strategis, are summarized in Table 2**.** The most used fractionation scheme was 35 Gy in 5 fractions (14 patients), and the mean prescribed BED_10_ was 69.4 Gy. The ratio of the prescription dose to the maximum PTV dose had a median of 89%, with interquartile range of 83% to 94%. The use of four dimensional (4D)-CT was elective, and it has been used in 40 (70.2%) out of the 47 patients. For 15 (26.3%) out of the 47 patients, there was no use of the 4D-CT. The information about 4D-CT was missing for two patients.

**Table 2:** Dosimetric variables. (Planning target volume: PTV).

**Variable**	
Dose (Gy) / Number of fractions (nb of lesions(%))	
● 24–28/3–5	3 (5.3)
● 30–35/3–5	27 (47.4)
● 40–45/4–5	10 (17.5)
● 50/5	9 (15.8)
● 50/10	8 (14.0)
Motion management strategy (nb of patients(%))	
● Free breathing	32 (56.1)
● Vaccm cushion (BodyFIX)	9 (15.8)
● Real-time tracking (Synchrony, X-sight)	6 (10.5)
● Abdominal compression	9 (3.5)
● Missing data	1 (1.8)
Prescription BED_10_ (Gy) (mean (SD))	69.4 (19.8)
ITV/GTV size (cc) (mean (SD))	27.0 (29.9)
ITV size (cc) (mean (SD))	28.9 (28.8)
GTV size (cc) (mean (SD))	25.8 (28.9)
PTV size (cc) (mean (SD))	71.7 (51.6)
ITV/GTV maximum BED_10_ (Gy) (mean (SD))	86.2 (30.7)
ITV/GTV minimum BED_10_ (Gy) (mean (SD))	60.9 (18.1)
ITV/GTV mean BED_10_ (Gy) (mean (SD))	77.5 (24.0)
PTV maximum BED_10_ (Gy) (mean (SD))	86.9 (32.8)
PTV minimum BED_10_ (Gy) (mean (SD))	43.0 (15.3)
PTV mean BED_10_ (Gy) (mean (SD))	74.1 (21.9)

### 3.2 Outcomes

**Fig. 1:**
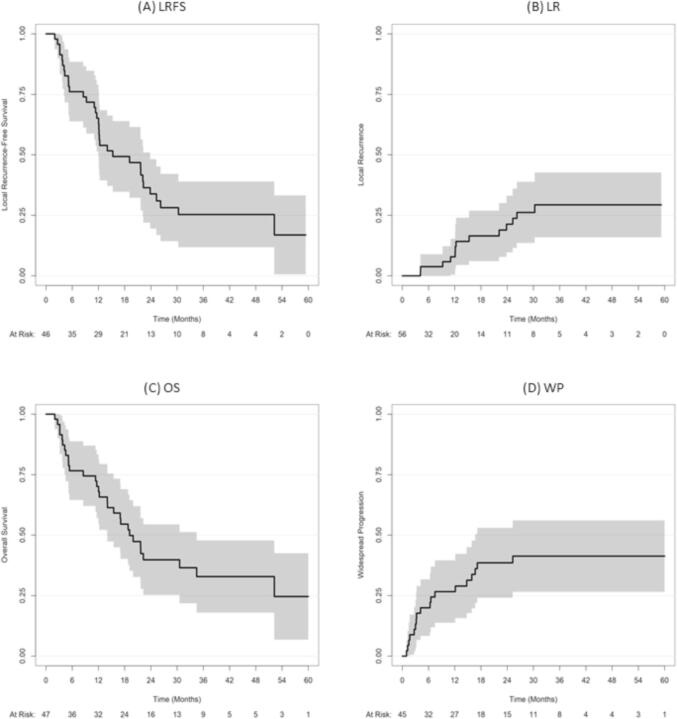
Kaplan-Meier graphs for LRFS (A) and OS (C) and competing risk analysis graphs for LR (B) and WP (D).

Results of Kaplan-Meier and competing risk analysis are featured in Table 3 and Fig. 1. The median LRFS, PFS, and OS were 15.3, 5.3, and 19.1 months, respectively.

**Table 3:** OS, LRFS, PFS, LR, and WP. (Confidence Interval: CI).

Time (months)	OS (95%CI)	LRFS (95%CI)	PFS(95%CI)	LR (95%CI)	WP (95%CI)
6	76.6% (65.4%-89.7%)	76.1% (64.7%-89.5%)	46.8% (34.5%-63.5%)	3.8% (0%-9%)	20% (8.2%-31.8%)
12	70.1% (58.2%-84.6%)	65.1% (52.7%-80.5%)	25.5% (15.7%-41.6%)	7.9% (0.3%-15.4%)	26.7% (13.6%-39.8%)
24	39.9% (27.6%-57.4%)	33.8% (22.1%-51.7%)	12.8% (6.05%-27%)	21.4% (9.3%-33.4%)	38.6% (23.9%-53.3%)
36	32.9% (20.9%-51.7%)	25.4% (14.9%-43.3%)	8.5% (3.3%-21.7%)	29.3% (15.5%-43.2%)	41.3% (26.3%-56.3%)
48	32.9% (20.9%-51.7%)	25.4% (14.9%-43.3%)	8.5% (3.3%-21.7%)	29.3% (15.5%-43.2%)	41.3% (26.3%-56.3%)

**Fig. 2:**
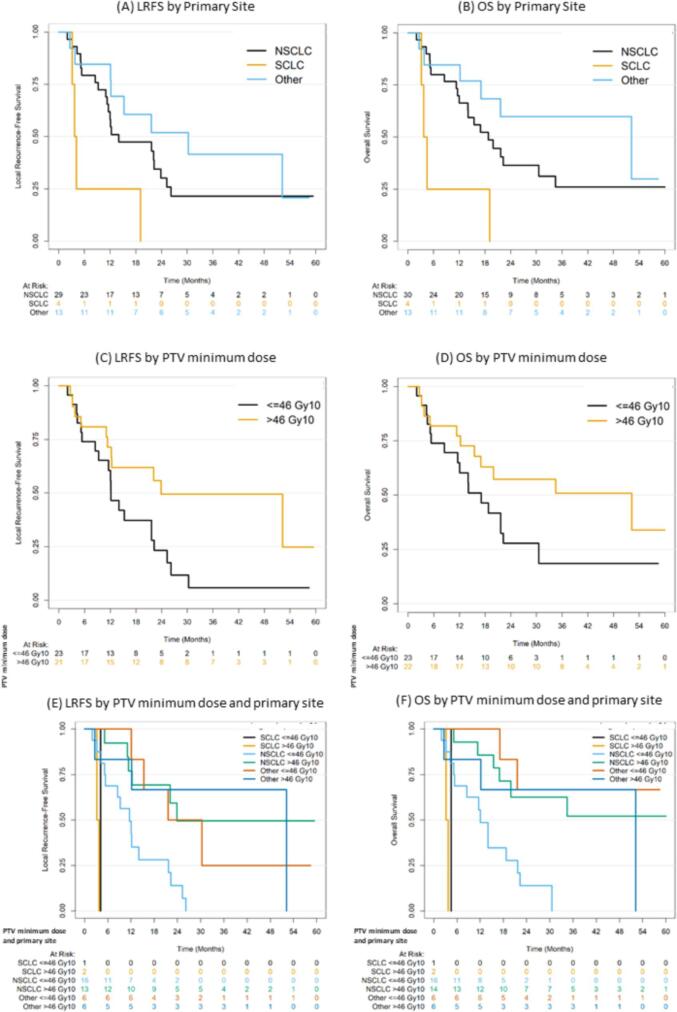
LR depending on BED10 (A) and WP depending on AMB (B) LRFS (A, C, and E) and OS (B, D, and F) depending on primary site and minimum PTV dose.

**Fig. 3:**
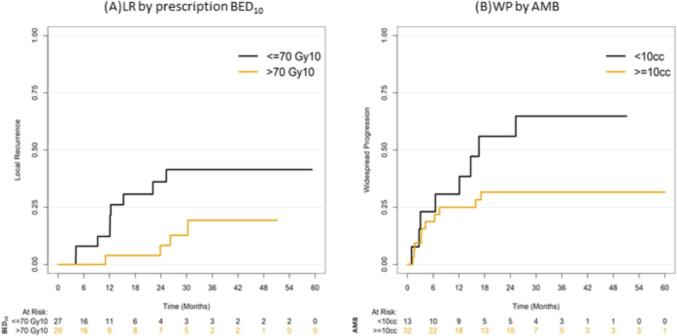
LR depending on BED10 (A) and WP depending on AMB (B).

On univariable analysis (UVA), primary small cell lung cancer (SCLC) was correlated with poor LRFS (p = 0.026), and a minimum PTV dose above a BED_10_ of 46 Gy (p = 0.061) was correlated with improved LRFS. This correlation remained statistically significant for SCLC (HR 15.3, 95%CI 3.8–61.4, p = 0.00012) and minimum PTV dose above a BED_10_ of 46 Gy (HR 0.37, 95%CI 0.18–0.76, p = 0.0064) on multivariable (MVA) analysis (Fig. 2A, 2C, 2E). For OS (Fig. 2B, 2D, 2F), a similar correlation pattern was observed for with SCLC (p-value: 0.017 on UVA and on MVA: HR 11.8, 95%CI 3.3–41.7, p = 0.00013) and minimum PTV dose above a BED_10_ of 46 Gy (p = 0.083 on UVA and on MVA: HR 0.42, 95%CI 0.2 – 0.9, p = 0.024). For LR, only a prescribed BED_10_>70 Gy (Fig. 3A) was an independent prognostic factor of a lower LR rate on MVA (HR 0.31, 95%CI 0.1–0.9, p = 0.039). For PFS, no statistically significant correlation was depicted on MVA. SCLC was associated with higher risk of WP on both UVA (p-value: 0.0022) and MVA (HR 7.23, 95%CI 2.6–5.4, p = 0.00045). The SCLC correlation remained significant for OS and LRFS when stratifying by the PTV minimum prescription dose. In addition, the trend towards worse WP with SCLC remained evident when stratifying by AMB (AMB). Interestingly, AMB>10 cc remained an independent predictor of a lower risk WP (Fig. 3B) compared with AMB less than 10 cc on MVA (HR 0.29, 95%CI 0.1–0.8, p = 0.017). Finally, only 1 (2.1%) patient developed an acute Grade 3 toxicity that consisted of abdominal pain, and no cases of adrenal insufficiency were recorded No treatment related Grade 4 or 5 toxicities were noted.

## 4 Discussion

In this multi-institutional study, we report a satisfactory local control and survival in oligometastatic patients treated with SBRT to their AGM, when a BED_10_ prescription dose of at least 70 Gy, and a minimum PTV dose above 46 Gy are achieved. Lung cancer, specifically NSCLC, was the most common primary site in our sample, and this observation is a direct reflection of cancer epidemiology in general and lung cancer pathophysiology in particular. Epidemiologically, lung cancer incidence is the second in males after prostate cancer [29], and in females after breast cancer [29]. In addition, it has a less indolent course than prostate cancer and is more prone for hematogeneous metastatic dissemination than breast cancer. As for the ubiquitous correlation of SCLC with poor outcomes compared to other primary sites, it is rather driven by the aggressive SCLC biology itself.

Compared to the previously published studies [18], our study is one of the largest published to date. More importantly, this study is among the first to report pooled results from multiple institutions, wherein uniform definitions of the oligometastatic state and SBRT fractionation are employed under a strict quality assurance process. These coherent inclusion criteria render our results well applicable to the oligometastatic patients, and more reflective than the preceding studies of the AGM outcomes in the OM patients’ population. For example, Zhao et al. reported the outcomes of 75 patients in total, the majority of which (54 patients, 72%) were treated with rather a palliative intention to a bulky AGM [19]. From the other hand, Chen et al. performed a systematic review of 39 studies with 1006 patients treated for AGM using SBRT, and reported a median biological equivalent dose (BED_10_,alpha/beta = 10) of 67 Gy, leading to a pooled one- and two-year local control (LC) rates of 82% and 63% respectively [18]. However, it was difficult to draw generalizable conclusions from the study due to two main factors. First, the definition of the oligometastatic state was inconsistent among the 39 studies. Second, the treatment scheme exhibited a wide range of heterogeneity with the delivered dose ranging between 8 and 60 Gy, and the number of fractions ranging between 1 and 27 [18]. In a more recent article, Buergy et al. published a multicenter analysis of 326 patients with 366 AGM, among whom 260 patients were treated with SBRT [20]. However, the included patients were a mix of metastatic states, as only 23.0% were considered oligometastatic and 24.5% had more than five lesions [20].

In comparison to surgical excision, SBRT has the advantage to address simultaneously multiple metastatic sites, with minimal interruption of the systemic therapy, via a safe and non-invasive procedure. The one- and two-year OS for SBRT in this study are 70.1%(95% CI, 65.489.7%) and 39.9% (95% CI, 27.6%-57.4%) respectively, and this is similar to the outcomes reported in previous studies where one- and two-year overall survival rates were 66% (95% CI, 58.2%-84.6%) and 42% (95% CI, 31%-53%), respectively [18]. The low rates of LR at two (7.9%, CI 0%–9%) and three years (21.4%, CI 9.3%–33.4%) suggest that SBRT can provide durable local control. The calculated three- and four-year OS of 32.9% (95% CI, 20.9%-51.7%) constitute a further evidence for the favorable prognosis of oligometastatic patients when treated with SBRT. The dynamics of SBRT association with improved OS are not fully explained yet, but multiple hypotheses have been suggested in the literature. Among these hypotheses, is the fact that the OM state is an intermediate status in the continuum spectrum of malignancy ranging from confined malignancy to widespread distant metastases. Under this hypothesis, ablative SBRT doses –similar to surgical resection- results in long-term disease control, delay the disease progression, and subsequently improve OS [30].

Despite the anatomical proximity of the adrenal gland to radiosensitive and critical structures such as the kidneys and the small bowel, and the strict use of non-palliative fraction schemes, the low incidence of Grade 3 toxicity in this study suggests that SBRT is a well-tolerated treatment modality for oligometastatic disease. In fact, the improved outcomes and reduced toxicity with SBRT nowadays has also been driven by the technological advancement in image guidance and tumor tracking. While older studies have used a large supero-inferior margins to the PTV in order to account for respiratory motion [31], the current practice using 4D CT and MR-Linac allows an accurate PTV targeting, without bypassing the organs at risk constraints. Given that various techniques for motion management have been used across centers and that ITV can be generated only when free breathing is used, a GTV/ITV poling was performed. Thus, BED_10_ dose were reported to the pooled GTV/ITV variable in the original database, and separate analysis cannot be conducted. While a dedicated GTV and ITV oriented analysis might be advantageous, the difference between GTV and ITV size was minimal (3.1) and GTV/ITV pooling was previously reported when performing across institutions SBRT analyss [32], [33].

From a dosimetric perspective, previous studies had established an association between high prescription dose and improved outcomes. For example, a strong positive association has been reported between SBRT dose and one- and two-year LC and two-year OS [18], with a BED_10_ of 60 Gy, 80 Gy, and 100 Gy predicting a one-year LC of 70.5%, 84.8%, and 92.9% and 2-year LC of 47.8%, 70.1%, and 85.6%, respectively [18]. In our study, a prescription dose of a BED_10_>70 Gy was associated with improved LC, but this association was not statistically significant for OS or LRFS. In addition, a much higher BED_10_ delivery to AGM may not be practical given proximity of critical organs, such as the stomach for left adrenal gland. Nevertheless, our analysis was the first to highlight the minimum PTV dose as a predictor of OS and LRFS. Under this perspective, the International Commission on Radiation Unit and Measurements (ICRU) 91 report on Prescribing, Recording, and Reporting of Stereotactic Treatments with Small Photon Beams [34] was published in 2017 and recommended the delineation of D98% and D2% for PTV > 2 cc in size. Our CORE database included patients treated between Janaury 1, 2008 and December 31, 2016 and the ICRU 91 nomenclature [34] could not have been adoped. Nevertheless, we think that minimum and maximum dose can serve as a good primary surrogate of dose distribution across patients and institutions. Moreover, our recommendation of a minimum PTV dose BED_10_ > 46 Gy would rather be reinforced (and not violated) if the point-dose constraint was replaced by the volume-dose constraints of the ICRU 91.

Despite the fact that the AGM lesion laterality was not available through this analysis, achieving a minimum dose of BED_10_ of 46 Gy to the PTV seems to be a practical requirement to implement in the future studies, and such requirement should be complemented by recommending a prescription dose of BED_10_ = 70 Gy, that is associated with a favorable local control. While prescription isodose lines and other heterogeneity parameters were not available from the study database, heterogeneity could be approximated by the ratio of the prescription dose to the PTC maximum dose. As for patients with treated simultaneously for more than two AGMs, the data on intentional dose reduction was not directly available through the study database, and such practice has likely differed by institution. Nonetheless, it seems that no major dose reduction was performed as prescription BED_10_ for those treated for a single AGM versus those treated with two or more AGMs.

This study is also the first to introduce the concept of AMB in order to account for the presence of multiple AGM in oligometastatic patients. Conceptually, a low volume of metastatic disease is expected to yield favorable outcomes, and this has been highlighted in few published studies. For example, Toesca et al. reported their single institution experience, and patients with AGM diameter less than 2.9 cm had a median OS of 54 months, compared with 11 months for those whom AGM diameter was higher or equal to 2.9 cm (p-value = 0.01) [35]. In contrast, our results are suggesting that a high AMB is a favorable prognostic factor for WP and this paradoxical association is not fully understood. One possible explanation emerges from the hypothetical possibility that SBRT enables better control of the overall disease in oligometastatic patients when OM are contained within one anatomical compartment (unilateral or bilateral adrenal glands), rather than multiple anatomical compartments.

Despite its multi-institutional nature, our study has certain limitations. First, it is a retrospective analysis that does not entail a direct comparison between SBRT and other therapeutic measures. In addition, our sample was dominated by lung cancer, and other primary sites such as breast and melanoma were underrepresented*.* As such, our analysis could not well account for the difference in prognosis among different sites, e.g. prostate cancer versus SCLC, and among different histologies of the same site, e.g. SCLC versus NSCLC. From the other hand, our database was representative of only four out of the nine Guckenberger et al. groups [22], the majority of which were either synchronous oligometastatic or metachronous oligorecurrence. Also, patients’ performance status was not available through this study database, though patient had likely a food performance status in order to be considered for SBRT course. Furthermore, the interplay of systemic therapy with the SBRT AGM course seems not to affect the outcomes in this study database. Given the retrospective nature of this manuscript, conclusions were exploited towards the dosimetric coverage, rather than the systemic therapy strategy that was likely heterogeneous across centers. Finally, grade 1 and 2 toxicities were not recorded.

In summary, our conclusions can be translated into the treatment strategy by including the minimum PTV BED_10_ > 46 Gy and prescription BED_10_ > 70 Gy in dosimetric guiding. Moreover, the study supports the delivery of therapeutic doses of SBRT even for patients with high AMB by including these patients in the treatment scheme with a definitive, non-palliative approach.

Given the limitations of this study, prospective studies are needed in order to further delineate further the clinical and dosimetric prognostic factors for AGM SBRT. Currently, multiple trials (NCT02759783 and NCT01761929) are conducted to compare the outcome of SBRT with the standard of care in patients with OM.

## 5 Conclusion

In the light of limited data availability for oligometastatic patients treated with SBRT for AGM, we present results of a large multi-institutional series. The results suggest that patients should be treated with a prescription BED_10_ dose of at least 70 Gy, and the minimum dose to the PTV should not be lower than 46 Gy. Currently, there is no evidence that a large AMB is associated with poor outcomes, and a high AMB should not preclude the administration of a definitive course of SBRT. The results of the ongoing prospective trials should contribute to the establishment of well-defined clinical eligibility criteria for SBRT, along a uniform consensus for dosimetric planning.

## Funding

Support for this study was provided as a grant from Elekta AB.

## Declaration of Competing Interest

Dr. Roi Dagan has an employment relationship with University of Florida. He receives compensation, remuneration, and/or funding from Elekta. Dr. Alexander V Louie has an employment relationship with Sunnybrook Health Sciences Center. He receives compensation, remuneration, and/or funding from AstraZeneca. Dr. Kristin J. Redmond has an employment relationship with Johns Hopkins University. She receives compensation, remuneration, and/or funding from Elekta, AstraZeneca, Accurav, and Biometrix. Dr. Sahgal receives compensation, remuneration, and/or funding from Elekta, Varian, BrainLAB, and VieCure. Dr. Tithi Biswas has an employment relationship with University Hospitals Seidman Cancer Center. She receives compensation, remuneration, and/or funding from Galera Therapeutics, AstraZeneca.
