# Supplementary material for: Outcomes and toxicities in oligometastatic patients treated with stereotactic body radiotherapy for adrenal gland metastases: A multi-institutional retrospective study
Source: Clin Transl Radiat Oncol. 2021 Oct 26;33:159–64. doi: 10.1016/j.ctro.2021.09.002 (PMC8885400; doi:10.1016/j.ctro.2021.09.002)
Supplement: Supplementary data 1 [file mmc1.docx]

**Appendix** - “Outcomes and Toxicities in Oligometastatic Patients Treated with Stereotactic Body Radiotherapy for Adrenal Gland Metastases: A Multi-Institutional Retrospective Study”

1. **Local Recurrence Over Time**

#### Univariable Regression Results

| Variable | HR | HR95Low | HR95High | Pvalue |
| --- | --- | --- | --- | --- |
| AgeAtSBRT (per unit) | 0.99 | 0.943 | 1.04 | 0.68 |
| Gender |  |  |  |  |
| F (Reference) | 1 |  |  |  |
| M | 1.59 | 0.556 | 4.53 | 0.39 |
| PrimarySite3Cat |  |  |  | Omnibus: |
|  |  |  |  | 0.908 |
| NSCLC (Reference) SCLC | 1  1.87 | 0.34 | 10.3 | 0.47 |
| Other Synch_Meta  Metachronous (Reference) | 1.09  1 | 0.32 | 3.68 | 0.9 |
| Synchronous | 0.972 | 0.291 | 3.24 | 0.96 |
| MetastaticInterval (per unit) | 1 | 0.997 | 1.01 | 0.24 |
| Bed (per unit) | 0.978 | 0.944 | 1.01 | 0.2 |
| Bed_Gr_Eq_60 (Y vs. N) | 0.333 | 0.114 | 0.975 | 0.045 |
| Bed_Gr_Eq_70 (Y vs. N) | 0.31 | 0.102 | 0.943 | 0.039 |
| Bed_Gr_Eq_80 (Y vs. N) | 0.892 | 0.259 | 3.07 | 0.86 |
| Bed_Gr_Eq_100 (Y vs. N) | 1.16 | 0.314 | 4.32 | 0.82 |
| ITV_GTV_Gr_Eq_10 (Y vs. N) | 3.32 | 0.742 | 14.9 | 0.12 |
| ITV_GTV_Max_Bed_Gr_Med (Y vs. N) | 0.678 | 0.233 | 1.97 | 0.47 |
| ITV_GTV_Min_Bed_Gr_Med (Y vs. N) | 0.522 | 0.183 | 1.49 | 0.23 |
| ITV_GTV_Mean_Bed_Gr_Med (Y vs. N) | 0.485 | 0.169 | 1.39 | 0.18 |
| PTV_Gr_Med (Y vs. N) | 2.6 | 0.806 | 8.39 | 0.11 |
| PTV_Max_Bed_Gr_Med (Y vs. N) | 0.678 | 0.233 | 1.97 | 0.47 |
| PTV_Min_Bed_Gr_Med (Y vs. N) | 0.427 | 0.134 | 1.35 | 0.15 |
| PTV_Mean_Bed_Gr_Med (Y vs. N) | 0.338 | 0.11 | 1.04 | 0.059 |
| PreSBRTSystemicTherapy (Y vs. N) | 0.556 | 0.156 | 1.99 | 0.37 |
| PostSBRTSystemicTherapy (Y vs. N) | 1.94 | 0.555 | 6.76 | 0.3 |

- 1. **Multivariable Model**

| Variable | HR | HR95Low | HR95High | Pvalue |
| --- | --- | --- | --- | --- |
| Bed_Gr_Eq_70 (Y vs. N) | 0.31 | 0.102 | 0.943 | 0.039 |

**2 Overall Survival**

#### Univariable Regression Results

| Variable | HR | HR95Low | HR95High | Pvalue |
| --- | --- | --- | --- | --- |
| AgeAtSBRT (per unit) | 1.02 | 0.986 | 1.05 | 0.29 |
| Gender |  |  |  |  |
| F (Reference) | 1 |  |  |  |
| M | 1.11 | 0.546 | 2.27 | 0.77 |
| PrimarySite3Cat |  |  |  | Omnibus: |
|  |  |  |  | 0.0301 |
| NSCLC (Reference) SCLC | 1  3.8 | 1.27 | 11.4 | 0.017 |
| Other Synch_Meta  Metachronous (Reference) | 0.549  1 | 0.224 | 1.35 | 0.19 |
| Synchronous | 0.588 | 0.258 | 1.34 | 0.21 |
| MetastaticInterval (per unit) TotalMetsNew | 0.993 | 0.981 | 1.01 | 0.32 |
| <=3 (Reference) | 1 |  |  |  |
| >3 | 1.23 | 0.417 | 3.63 | 0.71 |
| TotalMetsNew2 |  |  |  |  |
| 1 (Reference) | 1 |  |  |  |
| >=2 | 0.791 | 0.391 | 1.6 | 0.51 |
| Bed_mean (per unit) | 1 | 0.978 | 1.02 | 0.97 |
| Bed_mean_Gr_Eq_60 (Y vs. N) | 0.861 | 0.427 | 1.73 | 0.67 |
| Bed_mean_Gr_Eq_70 (Y vs. N) | 0.973 | 0.484 | 1.96 | 0.94 |
| Bed_mean_Gr_Eq_80 (Y vs. N) | 1.13 | 0.452 | 2.81 | 0.8 |
| Bed_mean_Gr_Eq_100 (Y vs. N) | 0.985 | 0.374 | 2.59 | 0.98 |
| ITV_GTV_sum_Gr_Med (Y vs. N) | 0.812 | 0.394 | 1.67 | 0.57 |
| ITV_GTV_sum_Gr_Eq_5 (Y vs. N) | 2.28 | 0.315 | 16.6 | 0.41 |
| ITV_GTV_sum_Gr_Eq_10 (Y vs. N) | 1.22 | 0.581 | 2.57 | 0.6 |
| ITV_GTV_Max_Bed_max_Gr_Med (Y vs. N) | 1.62 | 0.797 | 3.28 | 0.18 |
| ITV_GTV_Min_Bed_min_Gr_Med (Y vs. N) | 0.762 | 0.367 | 1.58 | 0.46 |
| ITV_GTV_Mean_Bed_mean_Gr_Med (Y vs. N) | 1.02 | 0.497 | 2.11 | 0.95 |
| PTV_sum_Gr_Med (Y vs. N) | 1.34 | 0.652 | 2.75 | 0.43 |
| PTV_Max_Bed_max_Gr_Med (Y vs. N) | 1.62 | 0.797 | 3.28 | 0.18 |
| PTV_Min_Bed_min_Gr_Med (Y vs. N) | 0.503 | 0.231 | 1.09 | 0.083 |
| PTV_Mean_Bed_mean_Gr_Med (Y vs. N) | 1.17 | 0.562 | 2.42 | 0.68 |
| PreSBRTSystemicTherapy (Y vs. N) | 0.797 | 0.362 | 1.75 | 0.57 |
| PostSBRTSystemicTherapy (per unit) | 0.849 | 0.322 | 2.24 | 0.74 |

#### Multivariable Model

Variable HR HR95Low HR95High Pvalue PrimarySite3Cat

NSCLC (Reference) 1

| SCLC | 11.8 | 3.34 | 41.7 | 0.00013 |
| --- | --- | --- | --- | --- |
| Other | 0.377 | 0.139 | 1.02 | 0.056 |
| PTV_Min_Bed_min_Gr_Med (Y vs. N) | 0.42 | 0.197 | 0.894 | 0.024 |

Summary of categories in “PTV_Min_Bed_min_Gr_Med”

Category Mininum of all PTV minimum delivered doses (BED, Gy10), minimum in category

<=Median 13.6 45.6

>Median 46.9 83.5

Mininum of all PTV minimum delivered doses (BED, Gy10), maximum in category

## **3 Local Recurrence-Free Survival**

#### Univariable Regression Results

| Variable | HR | HR95Low | HR95High | Pvalue |
| --- | --- | --- | --- | --- |
| AgeAtSBRT (per unit) | 1.01 | 0.986 | 1.04 | 0.34 |
| Gender |  |  |  |  |
| F (Reference) | 1 |  |  |  |
| M | 1.24 | 0.626 | 2.45 | 0.54 |
| PrimarySite3Cat |  |  |  | Omnibus: |
|  |  |  |  | 0.0453 |
| NSCLC (Reference) SCLC | 1  3.72 | 1.17 | 11.8 | 0.026 |
| Other Synch_Meta  Metachronous (Reference) | 0.634  1 | 0.285 | 1.41 | 0.26 |
| Synchronous | 0.525 | 0.218 | 1.27 | 0.15 |
| MetastaticInterval (per unit) TotalMetsNew | 0.999 | 0.993 | 1.01 | 0.8 |
| <=3 (Reference) | 1 |  |  |  |
| >3 | 0.969 | 0.315 | 2.99 | 0.96 |
| TotalMetsNew2 |  |  |  |  |
| 1 (Reference) | 1 |  |  |  |
| >=2 | 0.649 | 0.326 | 1.29 | 0.22 |
| Bed_mean (per unit) | 1 | 0.983 | 1.02 | 0.75 |
| Bed_mean_Gr_Eq_60 (Y vs. N) | 0.869 | 0.435 | 1.73 | 0.69 |
| Bed_mean_Gr_Eq_70 (Y vs. N) | 1.02 | 0.522 | 2 | 0.95 |
| Bed_mean_Gr_Eq_80 (Y vs. N) | 1.41 | 0.663 | 3 | 0.37 |
| Bed_mean_Gr_Eq_100 (Y vs. N) | 1.29 | 0.591 | 2.83 | 0.52 |
| ITV_GTV_sum_Gr_Med (Y vs. N) | 0.822 | 0.417 | 1.62 | 0.57 |
| ITV_GTV_sum_Gr_Eq_5 (Y vs. N) | 2.8 | 0.41 | 19.2 | 0.29 |
| ITV_GTV_sum_Gr_Eq_10 (Y vs. N) | 1.47 | 0.697 | 3.1 | 0.31 |
| ITV_GTV_Max_Bed_max_Gr_Med (Y vs. N) | 1.9 | 0.97 | 3.71 | 0.061 |
| ITV_GTV_Min_Bed_min_Gr_Med (Y vs. N) | 0.759 | 0.383 | 1.5 | 0.43 |
| ITV_GTV_Mean_Bed_mean_Gr_Med (Y vs. N) | 1.16 | 0.588 | 2.3 | 0.67 |
| PTV_sum_Gr_Med (Y vs. N) | 1.58 | 0.797 | 3.13 | 0.19 |
| PTV_Min_Bed_min_Gr_Med (Y vs. N) | 0.43 | 0.206 | 0.898 | 0.025 |
| PTV_Mean_Bed_mean_Gr_Med (Y vs. N) | 1.17 | 0.594 | 2.32 | 0.64 |
| PreSBRTSystemicTherapy (Y vs. N) | 0.803 | 0.394 | 1.64 | 0.55 |
| PostSBRTSystemicTherapy (per unit) | 0.999 | 0.424 | 2.35 | 1 |

#### Multivariable Model

Variable HR HR95Low HR95High Pvalue PrimarySite3Cat

NSCLC (Reference) 1

| SCLC | 15.3 | 3.79 | 61.4 | 0.00012 |
| --- | --- | --- | --- | --- |
| Other | 0.458 | 0.203 | 1.03 | 0.059 |
| PTV_Min_Bed_min_Gr_Med (Y vs. N) | 0.37 | 0.181 | 0.756 | 0.0064 |

Summary of categories in “PTV_Min_Bed_min_Gr_Med”

Category Mininum of all PTV minimum delivered doses (BED, Gy10), minimum in category

<=Median 13.6 45.6

>Median 46.9 83.5

Mininum of all PTV minimum delivered doses (BED, Gy10), maximum in category

1. Progression-Free Survival

#### Univariable Regression Results

| Variable | HR | HR95Low | HR95High | Pvalue |
| --- | --- | --- | --- | --- |
| AgeAtSBRT (per unit) | 0.989 | 0.961 | 1.02 | 0.46 |
| Gender |  |  |  |  |
| F (Reference) | 1 |  |  |  |
| M | 1.06 | 0.587 | 1.93 | 0.84 |
| PrimarySite3Cat |  |  |  | Omnibus: |
|  |  |  |  | 0.115 |
| NSCLC (Reference) SCLC | 1  3.88 | 1.75 | 8.62 | 0.00088 |
| Other Synch_Meta  Metachronous (Reference) | 1.17  1 | 0.578 | 2.36 | 0.67 |
| Synchronous | 0.766 | 0.389 | 1.51 | 0.44 |
| MetastaticInterval (per unit) | 0.997 | 0.99 | 1 | 0.33 |

TotalMetsNew

<=3 (Reference) 1

>3 1.59 0.757 3.35 0.22

TotalMetsNew2

1 (Reference) 1

| >=2 | 1.58 | 0.854 | 2.91 | 0.15 |
| --- | --- | --- | --- | --- |
| Bed_mean (per unit) | 0.996 | 0.98 | 1.01 | 0.67 |
| Bed_mean_Gr_Eq_60 (Y vs. N) | 0.961 | 0.542 | 1.7 | 0.89 |
| Bed_mean_Gr_Eq_70 (Y vs. N) | 1.2 | 0.665 | 2.16 | 0.55 |
| Bed_mean_Gr_Eq_80 (Y vs. N) | 0.844 | 0.4 | 1.78 | 0.66 |
| Bed_mean_Gr_Eq_100 (Y vs. N) | 0.824 | 0.365 | 1.86 | 0.64 |
| ITV_GTV_sum_Gr_Med (Y vs. N) | 0.834 | 0.46 | 1.51 | 0.55 |
| ITV_GTV_sum_Gr_Eq_5 (Y vs. N) | 0.801 | 0.409 | 1.57 | 0.52 |
| ITV_GTV_sum_Gr_Eq_10 (Y vs. N) | 0.93 | 0.544 | 1.59 | 0.79 |
| ITV_GTV_Max_Bed_max_Gr_Med (Y vs. N) | 1.61 | 0.882 | 2.95 | 0.12 |
| ITV_GTV_Min_Bed_min_Gr_Med (Y vs. N) | 1.1 | 0.608 | 2 | 0.75 |
| ITV_GTV_Mean_Bed_mean_Gr_Med (Y vs. N) | 1.13 | 0.619 | 2.06 | 0.69 |
| PTV_sum_Gr_Med (Y vs. N) | 1.29 | 0.691 | 2.42 | 0.42 |
| PTV_Min_Bed_min_Gr_Med (Y vs. N) | 0.709 | 0.39 | 1.29 | 0.26 |
| PTV_Mean_Bed_mean_Gr_Med (Y vs. N) | 1.08 | 0.597 | 1.97 | 0.79 |
| PreSBRTSystemicTherapy (Y vs. N) | 1.45 | 0.736 | 2.84 | 0.28 |
| PostSBRTSystemicTherapy (per unit) | 1.15 | 0.599 | 2.22 | 0.67 |

#### Multivariable Model

No significant predictors

1. Widespread Progression

#### Univariable Regression Results

| Variable | HR | HR95Low | HR95High | Pvalue |
| --- | --- | --- | --- | --- |
| AgeAtSBRT (per unit) | 1.02 | 0.986 | 1.06 | 0.25 |
| Gender |  |  |  |  |
| F (Reference) | 1 |  |  |  |
| M | 0.903 | 0.367 | 2.22 | 0.82 |
| PrimarySite3Cat |  |  |  | Omnibus: |
|  |  |  |  | 0.00779 |
| NSCLC (Reference) SCLC | 1  4.72 | 1.75 | 12.7 | 0.0022 |
| Other Synch_Meta  Metachronous (Reference) | 0.324  1 | 0.0694 | 1.52 | 0.15 |
| Synchronous | 1.02 | 0.381 | 2.73 | 0.97 |
| MetastaticInterval (per unit) TotalMetsNew | 0.99 | 0.965 | 1.01 | 0.42 |
| <=3 (Reference) | 1 |  |  |  |
| >3 | 4.92 | 2.32 | 10.4 | 3.1e-05 |
| TotalMetsNew2 |  |  |  |  |
| 1 (Reference) | 1 |  |  |  |
| >=2 | 1.63 | 0.657 | 4.04 | 0.29 |
| Bed_mean (per unit) | 1.01 | 0.985 | 1.03 | 0.5 |
| Bed_mean_Gr_Eq_60 (Y vs. N) | 1.86 | 0.692 | 4.99 | 0.22 |
| Bed_mean_Gr_Eq_70 (Y vs. N) | 1.91 | 0.744 | 4.92 | 0.18 |
| Bed_mean_Gr_Eq_80 (Y vs. N) | 1.58 | 0.552 | 4.51 | 0.4 |
| Bed_mean_Gr_Eq_100 (Y vs. N) | 1.31 | 0.407 | 4.22 | 0.65 |
| ITV_GTV_sum_Gr_Med (Y vs. N) | 0.603 | 0.239 | 1.52 | 0.28 |
| ITV_GTV_sum_Gr_Eq_5 (Y vs. N) | 0.488 | 0.128 | 1.87 | 0.29 |
| ITV_GTV_sum_Gr_Eq_10 (Y vs. N) | 0.436 | 0.18 | 1.05 | 0.066 |
| ITV_GTV_Max_Bed_max_Gr_Med (Y vs. N) | 1.36 | 0.542 | 3.43 | 0.51 |
| ITV_GTV_Min_Bed_min_Gr_Med (Y vs. N) | 2.14 | 0.818 | 5.58 | 0.12 |
| ITV_GTV_Mean_Bed_mean_Gr_Med (Y vs. N) | 1.21 | 0.479 | 3.04 | 0.69 |
| PTV_sum_Gr_Med (Y vs. N) | 0.8 | 0.323 | 1.98 | 0.63 |
| PTV_Max_Bed_max_Gr_Med (Y vs. N) | 1.36 | 0.542 | 3.43 | 0.51 |
| PTV_Min_Bed_min_Gr_Med (Y vs. N) | 0.935 | 0.368 | 2.37 | 0.89 |
| PTV_Mean_Bed_mean_Gr_Med (Y vs. N) | 1.58 | 0.624 | 3.98 | 0.34 |
| PreSBRTSystemicTherapy (Y vs. N) | 1.43 | 0.556 | 3.69 | 0.46 |
| PostSBRTSystemicTherapy (per unit) | 1.04 | 0.324 | 3.33 | 0.95 |

#### Multivariable Model

Variable HR HR95Low HR95High Pvalue

| PrimarySite3Cat NSCLC (Reference) SCLC | 1  7.23 | 2.39 | 21.8 | 0.00045 |
| --- | --- | --- | --- | --- |
| Other TotalMetsNew  <=3 (Reference)  >3 | 0.325  1  11.8 | 0.0813  2.57 | 1.3  54 | 0.11  0.0015 |
| ITV_GTV_sum_Gr_Eq_10 (Y vs. N) | 0.29 | 0.105 | 0.802 | 0.017 |
